# Beyond Basics: Can a Driving Simulator Reliably Reproduce Real Vehicle Dynamics?

**DOI:** 10.3390/s23218980

**Published:** 2023-11-05

**Authors:** Beatriz Fernandes, Eloisa Macedo, Jorge M. Bandeira

**Affiliations:** 1 TEMA—Centre for Mechanical Technology and Automation, Department of Mechanical Engineering, University of Aveiro, 3810-193 Aveiro, Portugal; bea.fernandes@ua.pt; 2 LASI—Intelligent Systems Associate Laboratory, 4800-058 Guimarães, Portugal

**Keywords:** driving behaviour, simulator vs. empirical tests, emissions

## Abstract

Despite constant technological innovation, road transport remains a significant source of pollutant emissions, and effective driver-behaviour changes can be considered as solutions that can increase the sustainability of road traffic in a short period. Thus, understanding driver behaviour plays a key role in assessing traffic-related impacts. Since real-world experiments entail some risks and are often not flexible, simulator-based experiments can be relevant to studying vehicle dynamics and driver behaviour. However, the reliability of the simulation results’ accuracy must be ensured. The primary objective of this paper is to present an exploratory analysis focused on the study of the reliability of a driving simulator to reproduce driving parameters that can then be used for emission estimation. For that purpose, tests were conducted by two drivers for urban and highway scenarios performed on a driving simulator and in real-world environments. Different road singularities composed events that were microscopically analysed. Second-by-second vehicle dynamic variables were recorded, and the pollutant emissions were estimated using the vehicle specific power (VSP) methodology. The results of this exploratory validation analysis showed that the total average emissions of all events were not significantly different (958.39 g for simulated and 998.06 g for empirical tests). Overall, the driving simulator can replicate vehicle dynamics from a microscopic perspective, especially for the urban scenario. This may be due to the more complex traffic conditions and road specificities that require more restrained driving behaviour. Nevertheless, VSP mode distributions did not follow the same pattern in 4 out of 10 events, meaning that the drivers displayed different behaviours in the simulated and empirical tests for those events. The relative errors range between 4 and 29% for carbon dioxide emissions and between 2 and 33% for nitrogen oxides emissions.

## 1. Introduction

The use of private vehicles is becoming more common each year, surpassing that of public transport [1], and currently, there are billions of vehicles on the roadways [2]. Despite ongoing technological advancements, road transport is still responsible for a number of problems that endanger both people and the environment. Nearly 65% of the consumption of worldwide oil-based products can be attributed to the transport sector [3]. Regarding emissions, road traffic is responsible for more than 20% of all greenhouse gas emissions in Europe [4], with carbon dioxide emissions rising yearly and significantly influencing climate change. Besides climate issues, road-transport-related emissions such as nitrogen oxides (NO_x_) have serious negative health effects [5]. In this context, it is crucial to pay attention to how to encourage sustainability in the operation of these vehicles to quickly and effectively cut off the negative impacts of road transport, especially regarding emissions, which is aligned with the net-zero target of the European Commission and the United Nations 2030 Agenda of Sustainable Development Goals (SDG 3, 11, and 13). Since real-world measurement campaigns are usually not easy to deploy, either because of the increased risk of collision or the unnecessary production of pollutant emissions, the use of a driving simulator can be considered a good approach for conducting experiments by simulating a driving environment to study vehicle dynamics and driver behaviour on a microscopic level. Driving simulators accurately simulate the driving experience without putting the driver or the environment at risk [6].

This exploratory analysis microscopically assesses how well a driving simulator might reflect the effects of vehicle emissions through data related to vehicle dynamics. This study aims to validate and compare the results of tests performed in both real-world and simulation environments. The real-world and simulation tests are designed to demonstrate not only the variations in vehicle emissions but also the variations in drivers’ behaviours, considering different types of routes. The driving simulator software and the On-Board Diagnosys (OBD) system coupled with the Global Navigation Satellite System (GNSS) provide instantaneous speed, acceleration, and position data for the vehicle, among other variables, under the simulation and real-world environments, which are carefully analysed. The research main contribution is to provide an evaluation of the extent to which a driving simulator can accurately reflect vehicle dynamics so that it allows for reasonable estimates of vehicle emissions. This allows us to understand driver behaviour variations and contribute to broader goals related to sustainability and policy development.

This paper is organized as follows: Section 2 provides an overview of the current state of the art by reviewing various papers that explore the advantages and applications of driving simulators as well as the validation of these devices. In Section 3, the materials and methods utilized in this study are described, including the collection of driving simulator and on-road data, case study scenarios, study participants, VSP and emission estimation, and validation. Section 4 presents and analyses the results obtained from the experiments conducted. Finally, Section 5 summarises the findings and draws conclusions regarding the ability of the driving simulator to accurately reproduce vehicle emissions.

## 2. Literature Review

### 2.1. Advantages and Applications of Driving Simulators

Driving simulator experiments present different advantages for research purposes. Driving simulators have good controllability, reproducibility, and standardisation and allow the possibility of simulating dangerous driving conditions without putting the driver at risk [7,8]. Regarding the data collection process, a driving simulator can measure performance accurately and efficiently, while it is far more complex to obtain complete, synchronized, and accurate measurement data from a real vehicle [7].

A considerable number of the scientific studies using these tools are framed in the clinical and psychological context [9,10]. A study conducted in 2021 used a driving simulator to determine reasonable speed limits for safety in dynamic low-visibility foggy conditions [11]. Participants completed trials with varying visibility and speed levels. A quantitative model was established and suggested speed limits were proposed based on visibility changes. The findings inform the development of variable speed limit (VSL) systems and reduce crash risks in foggy conditions with poor visibility. More recently, a study was conducted that emphasises the criticality of analysing driving behaviour to evaluate road safety, emissions, and fuel consumption [12]. The research takes into account variables like traffic conditions, road characteristics, and driving profiles. Through driving simulation tests, the study investigates how driver–road interaction influences gear-shift strategy, vehicle dynamics, safety indicators, comfort variables, and pollutant emissions. Performance assessment and database creation support in-depth analysis. Another research study that falls into this category is the 2018 study that aimed to address human errors and improve driver behaviour on curves by using different road-marking treatments [13]. Two treatments, optical circles and herringbone patterns, were tested in a driving simulator experiment on rural road sections. The study concluded that optical circles are effective for speed reduction and increasing driver attention, while herringbone patterns can help prevent head-on crashes by improving lateral position.

While the use of simulators has gained traction as a valuable tool in various domains, their effectiveness in reproducing real-world observable driving behaviour is not always demonstrated or evaluated. Therefore, validation is one of the most important topics to be studied in the driving simulation since the viability of all driving simulation studies depends on it.

### 2.2. Validation of Driving Simulators

Validity is the capacity of a simulator to simulate actual driving reliably [14]. The validity of driving simulators may be categorized into absolute validity and relative validity. Absolute validity is recognized when there are no statistical differences between driving behaviour measures observed in the simulated world and the real world [14,15].

The relative validity of the driving simulator should be assessed in situations when absolute validity is not achievable. Relative validity describes the extent to which the variation of a factor in a simulated world has an identical influence in the real world [16]. For instance, a research study can comprise 2 (driving simulator and real-world) × 2 (sober and drunk) designs for studying the behavioural change in the driving parameters. Here, researchers might find statistical differences in the numerical values observed in a real and a simulated world. However, relative validity can be achieved if the main effect of alcohol on different driving behaviour parameters shows similar effects in real and simulated worlds [14].

Meuleners and Fraser [17] focused on validating a laboratory-based driving simulator and provided early support for the relative validity of the driving simulator. The researchers recruited 47 drivers with valid licenses and instructed them to drive a specific route both on-road and in the driving simulator. The driving behaviours of the participants were assessed by an occupational therapist and two trained researchers using an assessment form. The results showed no statistical difference between the on-road assessment and the driving simulator for various driving behaviours, including mirror checking, observations, speed at intersections, maintaining speed, and obeying traffic lights and stop signs. These findings indicate that the driving simulator has relative validity and can be utilized for various road safety outcomes, providing a safer alternative to on-road testing and reducing the risk of harm to participants.

In [18], a general procedure was conducted for validating a driving simulation environment to analyse gap acceptance behaviour. The authors tested whether a synthetic indicator of gap acceptance behaviour showed significant differences when computed based on the simulated environment versus empirical observations. The proposed validation procedure was applied to the case of a three-leg roundabout. The results show that the mean critical gap estimated in the field and the mean critical gap estimated in the simulation environment were not significantly different. The proposed procedure can be applied in various contexts where gap acceptance behaviour is a central element in terms of the safety and operational performance of the traffic system under analysis.

Other studies were devoted to exploring the relevance of the vehicle specific power (VSP) mode distributions resulting from the empirical and simulated trips [19,20]. Zhao et al. developed an eco-driving feedback system using a driving simulator to enhance eco-driving training [20]. The system provides real-time feedback and voice prompts during driving to improve drivers’ eco-friendly behaviour. After driving, participants receive an evaluation report with fuel consumption rank, potential fuel savings, and personalized driving advice. The researchers used a microscopic emissions model based on VSP distribution to calculate the emissions on the empirical trips. In testing, the system led to a 5.37% reduction in CO_2_ emissions and a 5.45% decrease in fuel consumption. These findings highlight the system’s effectiveness in promoting eco-driving behaviour, resulting in lower emissions and fuel usage. In 2016, Yu et al. proposed to test and validate the feasibility and applicability of a driving simulator approach in generating vehicle activity data to produce VSP values and then to estimate emissions [19]. The study concluded that the driving simulator can be considered a useful test tool for estimating vehicle emissions, particularly for scenarios where the driving time is relatively short and the network and traffic conditions are less complex. Based on the literature review, we can conclude that research on the validation of both vehicle dynamics and emission estimation is rather scarce.

The present paper aims to develop an exploratory analysis of potential ways of validating the use of a driving simulator in environmental assessment studies. The main contributions of this study are as follows:

- Analysing driving behavioural differences in urban and highway environments;
- Comparing emissions outputs between micro event-based and route-level analysis;
- Analysing distributions of simulated and real-world VSP modes and the impact of these distributions on the estimation of a local air pollutant (NO_x_) and a GHG (CO_2_).

This research expects to expand the scientific understanding of driving simulator-related validation studies with higher goals focused on the estimation of the environmental impacts of different driving behaviours by exploring patterns under an urban and a highway scenario. The findings are valuable in understanding how drivers’ responses and actions vary in different environments for both empirical and simulation contexts and in showing the potential application of using data collected from driving simulators, since they prove in general, to be valid.

## 3. Materials and Methods

**Figure 1:**
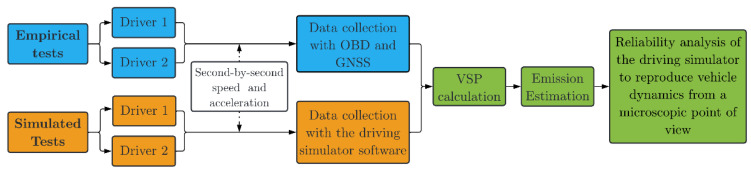
Research process framework.

Figure 1 shows the overall research process framework. This research is based on two distinct groups of data, one from the simulated testing and the other from the empirical tests. Both tests were performed by two different drivers, and data related to vehicle dynamics were recorded at 1 Hz frequency. A microscopic emission model estimation approach based on the vehicle specific power (VSP) model was used to estimate emissions of a local pollutant (NO_x_) and greenhouse gas (CO_2_). Finally, different methods to test the accuracy of the driving simulator in simulating vehicle emissions were explored.

### 3.1. Driving Simulator Data Collection

The experiments were carried out in a simulation environment using Carnetsoft research simulation software V8.9 [21]. The setup contained a multimonitor display (left, centre, and right), a monitor for data processing, and a Logitech G29 steering wheel, shifter, and pedals. Two cameras were also included to record each (on-road or simulation) scenario and allowed for checking all traffic conditions, being synchronized with the simulator. Over each run, drivers were monitored with basic questions to spot any potential indicators of simulator sickness or exhaustion. Instantaneous data of relevant variables, such as speed, acceleration, time, and location coordinates were collected and converted to an Excel spreadsheet.

### 3.2. On-Road Data Collection

A light-duty diesel (EURO VI) vehicle was used to match the characteristics of the simulated environment. It was equipped with an on-board diagnostics (OBD) device (OBDII-ELM), and a GNSS data logger was used to collect second-by-second vehicle dynamics and internal engine data such as voltage, fuel level, engine load, and engine speed. The device was connected to the vehicle and then synchronized with the “TORQUE” mobile app over Wi-Fi to provide access to vehicle performance information. This mobile app also continuously recorded the location of the car. All devices were properly calibrated and synchronized in each experiment. The retrieved information was saved as an Excel spreadsheet. The research team already developed emission factors for this vehicle type in a previous study [22] through several on-road emission measurements made with a portable emissions measurement system (PEMS)—a 3DATX ParSYNC integrated PEMS. The vehicle was also equipped with video cameras to monitor traffic-related conditions that may yield some constraints on the tests (e.g., engine shutdown, congestion, abnormal traffic volume).

### 3.3. Microscopic Analysis: Selection of Events and Critical Distances

Infrastructure singularities and traffic-related conditions influence driving behaviour and patterns. An overall route-level analysis conducted based on empirical or simulation data may yield misrepresentations of the traffic-related impacts, generating, e.g., over- or underestimation errors in the estimated emissions, and thus, may lead to erroneous conclusions. This is particularly relevant in the case of simulation experiments since specific outcomes can be masked and the capability of the simulator to reproduce accurate driving dynamics necessary for detailed emissions impact assessment can be unreliable. The microscopic analysis provided here involves an assessment of specific events that often occur in a road network, in particular, related to urban and highway scenarios. Based on scenarios available in the simulator, we chose those as reported in Table 1. In both empirical and simulation experiments, we tried to reproduce similar events so that a comparative evaluation could be carried out. The goal of the current study is to examine the driver’s behaviour while approaching and exiting areas of these events. In the Urban 1 event, drivers have priority but often check for oncoming traffic prior to proceeding with the right-turn manoeuvre, while in the Urban 2 event, drivers should always yield to oncoming traffic. In the Urban 3 and 4 events, drivers must comply with the traffic light and then proceed with the manoeuvres. In the Urban 5 and 6 events, drivers must stop the vehicle and then safely proceed with the right- and left-turn manoeuvres, respectively. The Urban 7 event corresponds to the situation where drivers face yield signs or an uncontrolled intersection. Next, concerning the events selected to evaluate the driving behaviour on highways, the Highway 8 event forces drivers to enter the highway from an on-ramp and merge with the traffic flow, while the Highway 9 event corresponds to the case of using the off-ramp for leaving the highway. Finally, the Highway 10 event allows drivers to freely and safely drive without the need for executing any other manoeuvres. These were chosen to allow for an evaluation of different driving behaviour settings, such as the response of drivers to traffic light signs or crosswalks, how smoothly or not the manoeuvres are executed, how drivers navigate along the event, and the drivers’ ability to adjust speed. For these experiments, there was the need to select the influence area associated with each event and discard the potential influence between events. Thus, based on the variable related to distance to the intersection (collected from the real vehicle position and the simulator), the focus should be related to the driver perception combined with the driving behavioural profile change in the approaching and exiting areas of an event. Two considerations were made to set the critical distances: (1) the minimum distance that was necessary to observe changes in driving behaviour before and after the event, such as the different pattern of the speed profile; and (2) the maximum distance was constrained so that the influence of other events or upstream or downstream singularities was avoided. Selected critical distances are also reported in Table 1 for each type of event. In this study, each driver performed four routes—two simulated and two real-world trips concerning the highway and urban scenarios, which included six runs. It should be emphasised that the aim of this study is not to validate a driving simulator in different events (a much more representative sample would be required) but to explore validation metrics and identify trends and major sources of error that should be considered in future research studies.

### 3.4. Case Study Scenarios

Two scenarios were chosen from the Carnetsoft software V8.9 [21]: an urban road and a highway.

The roads in the simulation accurately reflect the real-world context; they are surrounded by buildings and included different road agents, such as vulnerable road users. The road singularities included traffic signals, speed restrictions, and speed bumps. Regarding the highway simulation experiment, speed limits (as in real trips), various entrances and exits, and long distances to move forward were included.

**Figure 2:**
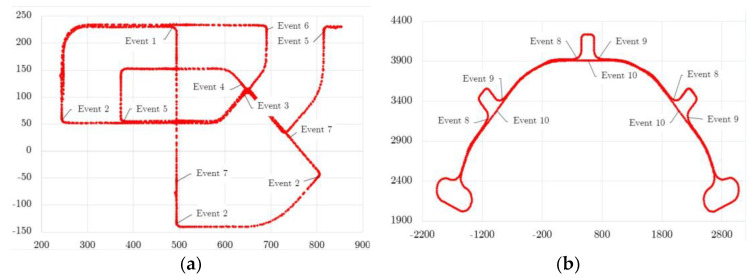
Maps of the simulation trips and tagged events. (**a**) City map. (**b**) Highway map.

**Figure 3:**
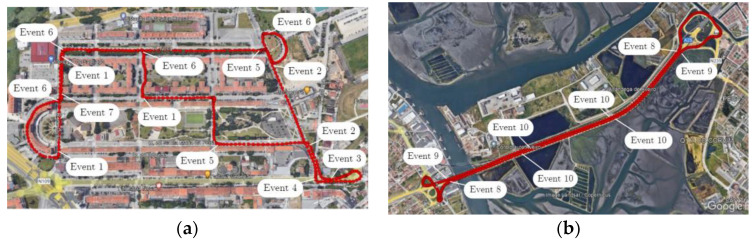
Maps of the empirical trips and tagged events. (**a**) City map. (**b**) Highway map.

It was crucial that the empirical scenarios matched the simulated ones as closely as possible from the point of view of the characteristics of the infrastructure, traffic context, urban environment, and weather conditions. For that purpose, the city of Aveiro, near the University of Aveiro campus, was chosen for conducting all empirical tests. Tests were carried out during daylight with sunny weather. There was no significant traffic congestion in any of the on-road experiments; thus, the simulator environment was set to reflect similar traffic conditions. The following figures display the simulation (Figure 2) and empirical (Figure 3) maps with the tagged events, as mentioned in Table 1, that were used as routes for conducting the experiments.

Although the real routes do not coincide with the simulator scenario, the research team designed a route that involved all the different events that are present in the simulation environment. Thus, we end up with different numbers of repetitions of some of the events, as reported in Table 1. The analysis will focus on exploring the different driving behaviour profiles within the critical areas of each event from either the point of view of dynamics and operation or the associated emissions.

### 3.5. Study Participants

Two volunteers participated in the study by conducting experiments in both simulated and real-world settings. They were asked to drive as they typically would, and training sessions were held to help them become familiar with the simulator. The drivers had similar levels of driving experience, but Driver 2 (a male) had never driven in the city of Aveiro and was not familiar with its traffic context.

### 3.6. VSP and Emission Estimation

In this study, the emissions were estimated using a microscopic model. The vehicle specific power (VSP) model was used here since it enables second-by-second estimation of pollutants by using data on vehicle speed and acceleration [23]. VSP is an estimate of the power demand on the engine during driving. It is an effective parameter for estimating vehicle emissions since it can be directly interpreted physically and has a strong statistical correlation with vehicle emissions [24].

The *VSP* value for light-duty vehicles on flat roads is given by [25]:

(1) $$VSP=v\;\cdot \left(1.1\;\cdot \;a+0.132\right)+0.000302\;\cdot \;v^{3},$$

where *VSP* is the vehicle specific power (kW/ton); *v* is the instantaneous vehicle speed (m/s); and *a* is the instantaneous vehicle acceleration (m/s^2^).

According to [26], a 14-mode VSP-based binning approach is suitable for estimating the operating modes of a light-duty (diesel) vehicle. Each operating mode corresponds to an emission factor that can then be used to calculate the emissions produced during driving. The VSP methodology ensures the following: (1) each mode should have an average emission rate statistically significantly different from any other mode, and (2) no single mode should predominate the estimation of total emissions for a typical trip. The four driving modes—idle, acceleration, cruise, and deceleration—can be easily represented using this methodology.

For each second of driving, a VSP value is assigned to a VSP mode, which in turn corresponds to an average of emissions, providing an emission factor for each bin. In this study, we focused on estimating instantaneous NO_x_ and CO_2_ emissions (g/s). Total emissions generated during each event are estimated according to

(2) $$E_{p}=\overset{14}{\underset{i=1}{\sum }}t_{VSP_{i}}\cdot ef_{p_{i}}$$

where *E_p_* is the emission of pollutant *p* (NO_x_ or CO_2_) generated in each event; $t_{VSP_{i}}$ is the time spent (sec) in each VSP mode *i*; and $ef_{p_{i}}$ is the emission factor (g/s) for pollutant *p* associated with each VSP mode *i*.

In terms of VSP modes, the following conclusions can be drawn: (1) VSP mode 1 represents strong decelerations; (2) VSP mode 2 represents small decelerations; (3) VSP mode 3 occurs when the vehicle is stationary, in idle condition, or when it starts to move; and (4) when the vehicle accelerates, the VSP mode value can range from 4 to 14; this value is proportional to the values of acceleration and speed; in general, the faster the car moves and the harder it accelerates, the larger the VSP mode is.

### 3.7. Validation

This study’s primary goal is to explore methods to assess the capability of a driving simulator to replicate real driving conditions for emission estimation. The analysis approach followed in the present study is mainly concerned with providing a comparative evaluation based on the following:

- Simulated vs. observed travel time;
- Speed and acceleration distribution of observed and empirical data (e.g., maximum, minimum, and average);
- Testing to find out whether the VSP mode distributions are different;
- Estimation of CO_2_ and NO_x_ emissions.

To make conclusions that support or not the evidence of reliable results provided by the experiments conducted under the driving simulator, the empirical and simulated VSP mode values were validated using the two-sample Kolmogorov–Smirnov (K-S) test for a 95% confidence level. This is a nonparametric goodness-of-fit test and is used to determine whether two distributions differ. This step aims to determine whether or not drivers respond similarly while operating a vehicle on a real road versus on a driving simulator under identical driving conditions [27].

The K-S test is one of the most practical and comprehensive nonparametric methods for comparing two samples because it is sensitive to variations in the location and form of the cumulative distribution functions of the two samples [28]. This test evaluates the discrepancy between the estimated and observed VSP mode distributions by quantifying the distance between the empirical and the cumulative distribution functions of two samples [28]. This research applied this test for both drivers for the VSP mode average frequency distribution of each event. The null distribution of the two-sample K–S test is calculated under the null hypothesis that the empirical and simulated VSP modes are drawn from the same distribution [29]; this hypothesis is then rejected or not after the following steps:

1. The cumulative probability of each VSP mode is calculated for the simulated and empirical results;
2. The maximum difference between simulated and empirical cumulative probability is discovered (*D*-*value*);
3. The *D*-*critical* is found using the following equation (Equation (3)):

(3) $$D_{critical}^{S}=1.36\sqrt{\frac{n+m}{nm}}$$

where *n* and *m* are the empirical and simulated sample sizes, respectively.

The sample size for each event corresponds to the sum of all VSP mode frequencies in each test. The null hypothesis is rejected if the *D*-*value* is greater than the *D*-*critical* value because it shows that no sample follows the same distribution and that the driver does not behave consistently in both simulated and real-world tests.

## 4. Results

Firstly, after performing an outlier detection to discard nonrelevant data from both experiments, a descriptive analysis of dynamic variables for the various simulated events is carried out for both drivers. Secondly, the values of estimated emissions from kinematic data obtained in the real environment and the simulator are compared. Finally, the analysis focuses on selected specific events, namely, the distribution of VPS modes and box plots of acceleration and speed data, to explain differences and similarities between driving profiles in the simulator and the real environment.

### 4.1. Participants’ Driving Behaviour

Table 2 reports the obtained results of speed, acceleration, and deceleration for all events and drivers, namely minimum (Min), lower quartile (Q1), median (Q2), upper quartile (Q3), maximum (Max), and average values. The main goal is to enable a descriptive analysis of relevant factors linked to driver behaviour in both environments.

These results show higher maximum and average speeds in the simulator, which is aligned with the findings reported in previous studies (e.g., [30,31]). A possible reason for this outcome is that drivers are less cautious about speeding up in virtual environments than in real-world conditions [19]. From the values in Table 2, it is also clear that Driver 1 typically breaks harder in the simulator than on the real road. The fact that the simulator’s brake pedal is more sensitive than an in-vehicle pedal may be a plausible explanation for this deviation. According to [19], the speed fluctuation in a real-world experiment would be excessive if the driver used the same pedal pressure to regulate the brake. Another explanation may be the lack of movement stimuli in static driving simulators such as the one used in this study.

### 4.2. Estimation of Emissions per Event in the Real-World and Simulated Environments

Table 3 displays the average CO_2_ and NO_x_ emissions estimated through the VSP methodology based on instantaneous speed and acceleration data collected in simulated and real urban environments. It also provides the generated average emissions. The coefficients between drivers’ emissions reflect the relationship between them in simulated and real environments. Values closer to 1 reflect more similar driver behaviour in both environments. The relative error between simulated and empirical emissions is also calculated. Lower relative error values suggest the capability of the simulator to reflect the absolute emissions generated in the real environment.

Table 3 shows that the events “right-turn with priority”, “right-turn after a stop sign”, and “left-turn after a stop sign” had the lowest errors between empirical and simulated emissions (10–13%). On the other hand, “turn left without priority” and “give way” are the events in which the observed relative errors were higher (up to 33%). Section 4.3 provides details on VSP distributions on these traffic events to better understand such differences.

It is also important to emphasise that in four of the seven urban events, the simulator data led to an underestimation of emissions compared to the kinematic data observed in an urban environment. However, in the event of “right-turn after a traffic light”, there were cases of both overestimation and underestimation, depending on the driver. In two urban traffic situations, namely, “left-turn without priority” and “give way”, there was an overestimation of the simulator. This variability reinforces the importance of analysing the simulator’s performance based on exploring event outcomes at a microscopic level and avoiding the occurrence of misleading validation and aggregation based on absolute errors on the whole trip length. Considering now the highway environment, Table 4 reports the obtained estimates for the emissions for both drivers and each different event.

While in the events “moving forward” and “exiting highway” the kinematic data from the simulator led to an overestimation of NO_x_ and CO_2_ emissions (2–18%), a clear underestimation of emissions (up to 20%) was found in the event “entering highway”. Despite these differences in absolute values, interestingly, the data resulting from the associated emissions ratio suggest a high capability of the simulator in representing behavioural variations of the two analysed driving outcomes.

Finally, aggregated data from the highway show that very small errors of CO_2_ emissions estimated from observed and empirical data (≈1%) can result from cancelling errors.

### 4.3. Speed, Acceleration, and VSP Distributions

A thorough analysis is presented of the events where the differences between the emissions estimated by the simulator data and the OBD device were the largest or the smallest in urban and highway environments.

#### 4.3.1. Left Turn without Priority

This event characterises the movements when the driver turns left without priority because of a yield sign or the driving rules. Depending on the existing traffic flow intensity, the driver can be required to slow down or even stop the vehicle. This event’s analysis covers 30 m before and 30 m after the intersection midpoint.

**Figure 4:**
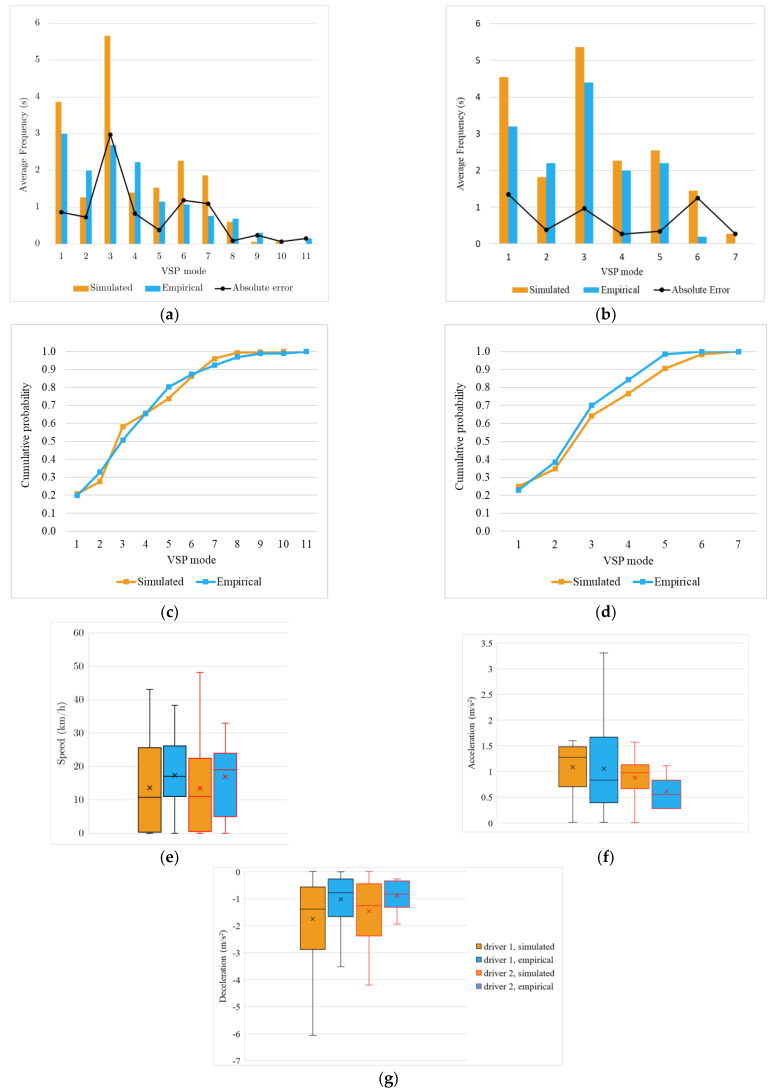
Left turn without priority event results: average of time spent in each VSP mode and absolute error between simulated and empirical values for Driver 1 (**a**) and Driver 2 (**b**). Cumulative distributions of simulated and empirical VSP modes for Driver 1 (**c**) and Driver 2 (**d**). Box plot for speed (**e**), acceleration (**f**), and deceleration (**g**).

Figure 4a,b show the time spent in each VSP mode for each driver in real and simulated environments. The first note is the higher discrepancy in mode 3 associated with idling situations. The second note is that although this event has recorded the largest difference in estimated emissions (30%), data shows that with the exception of mode 3 and Driver 1, the VSP mode distribution follows a similar trend for both drivers with an absolute error virtually lower than 2 s for any mode. The shape of the empirical and simulated cumulative distribution of VSP modes (Figure 4c,d), also supported by the K-S test results presented in Section 4.4, suggest that the frequencies of the VSP modes in the real-world and simulated tests are derived from the same distribution. The speed box plot (Figure 4e) shows that the bulk of empirical and simulated data is in the same range. However, there is an asymmetry in the empirical data for higher values, making the travel time slightly shorter. With different levels of variability, the acceleration and deceleration plots confirm a tendency for smoother accelerations in the empirical tests.

Overall, these data suggest that the differences in emissions estimated from empirical and simulated data are more related to specific road traffic circumstances and slightly longer travel times than to different driving behaviours of drivers in the real and simulated environments. Therefore, the ability of the model to reproduce the driving behaviour of drivers should not be ruled out despite the considerable errors in emissions reported in Table 3.

#### 4.3.2. Right Turn with Priority

This event occurs when the driver changes direction to the right without deferring to another vehicle. The event is examined along 60 m, i.e., 30 m before and 30 m after the intersection midpoint.

**Figure 5:**
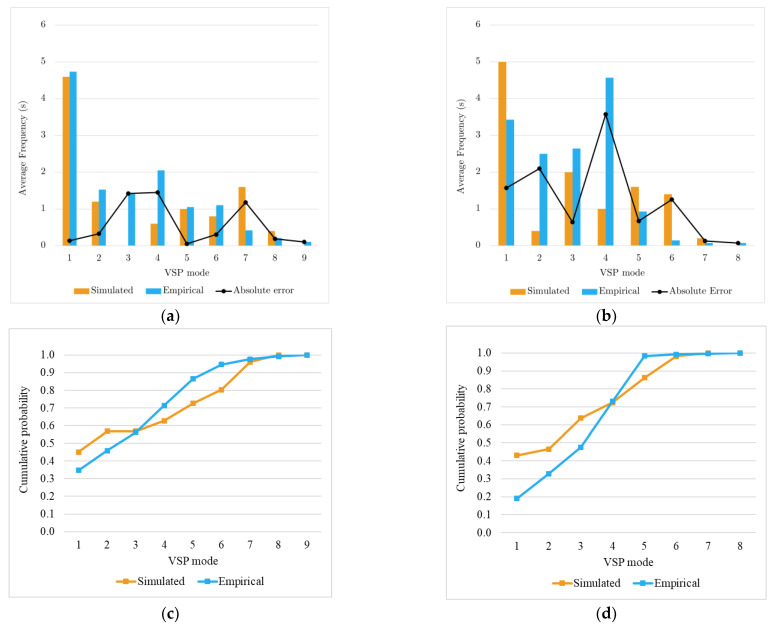

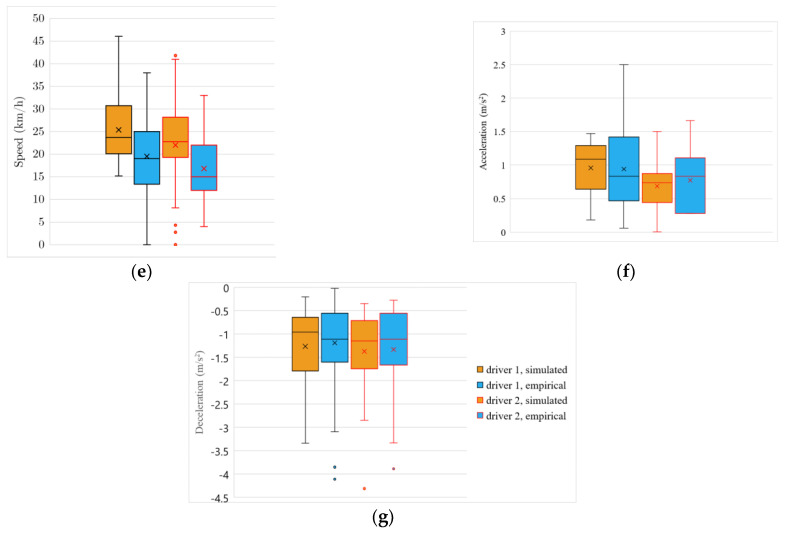
Right turn with priority event results: average of time spent in each VSP mode and absolute error between simulated and empirical values for Driver 1 (**a**) and Driver 2 (**b**). Cumulative distributions of simulated and empirical VSP modes for Driver 1 (**c**) and Driver 2 (**d**). Box plot for speed (**e**), acceleration (**f**), and deceleration (**g**).

Figure 5a,b show a significant difference in the average frequency of mode 4, especially for Driver 2. This can be explained by the fact that the driver travelled at lower speeds during the on-road trips (average of ≈17 km/h) compared to the simulated trips (average of ≈22 km/h), as shown in Figure 5e. Figure 5d further supports this evidence, In the empirical tests, Driver 2 virtually never generated a VSP mode greater than 5.

Through visual analysis of the graph (Figure 5c,d) and supported by the K-S test data (Section 4.4), it is evident that there is a greater similarity between the distributions of the real-world and simulated VSP modes for Driver 1 than Driver 2. In the empirical tests, Driver 2 never generated a VSP mode greater than 5. It is also possible to see from Figure 5b that, for Driver 2, the difference between the simulated averages of VSP modes 1 and 2 is significantly larger than the empirical difference between these modes.

Depending on the speed and acceleration, VSP mode values can change considerably. Therefore, this does not necessarily imply that the driver deceleration values are higher in the simulator than on the real road. In the simulation, the acceleration box plot (Figure 5f) is shorter for both drivers. The decrease in variability in the simulated environment may be related to more sources of randomness in real traffic contexts as opposed to the simulated environment, where the degree of predictability was higher.

Overall, it is important to note that although the differences in the VSP modes are distinct for both drivers, the relative error in calculating emissions from simulated and empirical data is similar (11–13%) for both drivers. However, for Driver 1, the small difference in emissions estimates is associated with a similar distribution of kinematic data, while for Driver 2, the similarity in emissions estimates is built from the cancellation of overestimations and underestimations of some VSP modes.

#### 4.3.3. Moving Forward

This event occurs throughout 800 m, selected from the section of the route between the highway entrance and exiting and not affected by the interchange traffic flows.

**Figure 6:**
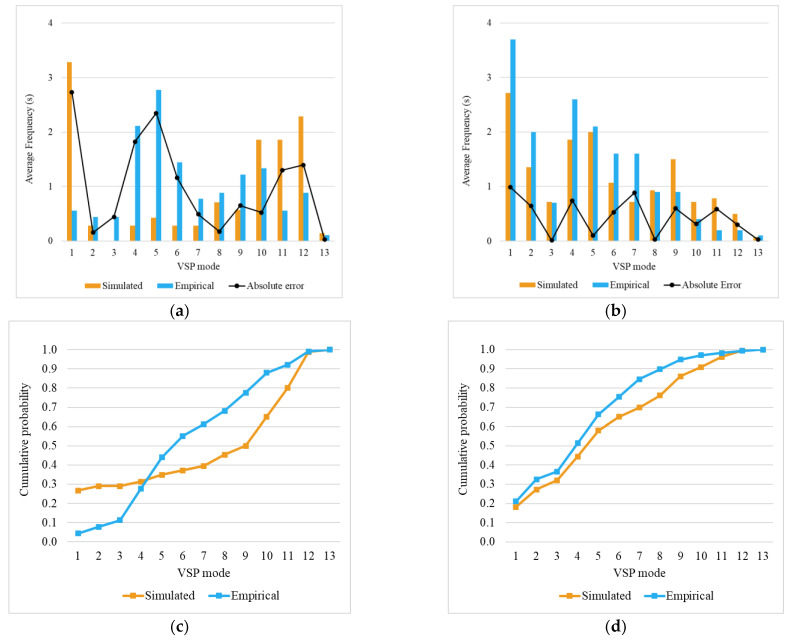

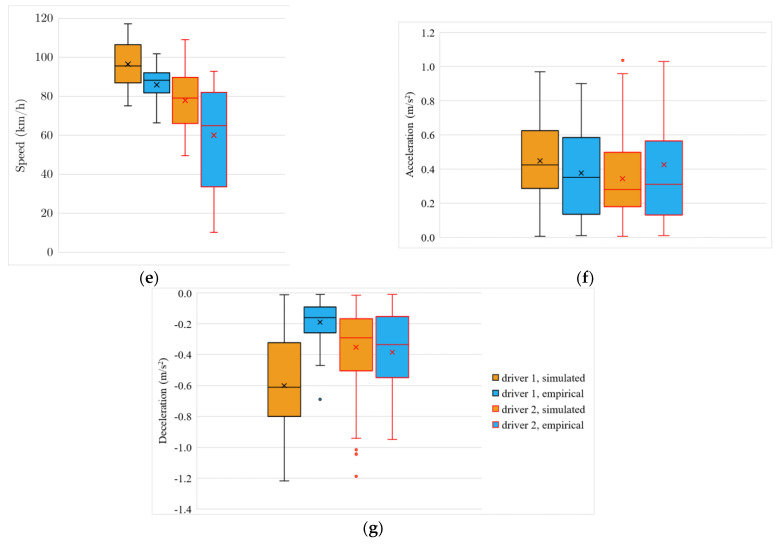
Moving forward event results: average of time spent in each VSP mode and absolute error between simulated and empirical values for Driver 1 (**a**) and Driver 2 (**b**). Cumulative distributions of simulated and empirical VSP modes for Driver 1 (**c**) and Driver 2 (**d**). Box plot for speed (**e**), acceleration (**f**), and deceleration (**g**).

In this event, there is a high discrepancy in the distribution of simulated and observed VSP modes, particularly noticeable for Driver 1’s VSP modal frequency cumulative distribution (Figure 6c) and confirmed by the results of the K-S test. Modal bins 4, 5, and 6 frequently appear throughout Driver 1’s empirical trials. GNSS tracking data and video cameras showed that this mode’s frequency was caused by slight decelerations while moving at high speed. This situation, which is quite frequent in highway driving, results from the appearance of traffic with a slower speed ahead of the vehicle being analysed. Driver 2 exhibits a comparable pattern of accelerations and decelerations between the simulator and empirical environments compared to Driver 1. Figure 6f,g demonstrate how similar the accelerations and decelerations are, but the speed as shown in Figure 6e was higher and more consistent throughout the tests in the driving simulator. The slight variations in VSP modes depicted in Figure 6b would be explained by this. The differences between the VSP distributions of the two conductors partially explain the difference in the order of magnitude of the relative errors reported in Table 4—16% emissions for Driver 1 and 2% emissions for Driver 2.

Acceleration box plot graphs show that Driver 1 decelerated more abruptly in the virtual tests and accelerated more vigorously in the empirical ones, as shown in Figure 6f and 6g, respectively. Overall speed (Figure 6e) remained constant for both tests but was higher in the virtual setting. The high deceleration levels in the simulated tests are thought to be caused by traffic. The high frequency of modal bin 1 in the experiments (Figure 6a,b) could also be explained by the driver’s risk perception and the need to adapt his speed to traffic flow.

Although the estimated emissions output for empirical and simulator kinetic data are similar (error > 2%), it should be mentioned that in the case of Driver 2, the similarity in emissions comes from a different distribution of VSP modes with slightly longer travel time and more time spent in low modes in the real environment than in the simulated one.

#### 4.3.4. Entering Highway

This section covers 150 m from the highway access slip road and 150 m from the start of the acceleration lane. In both situations, the volume of traffic on the highway is relatively high, requiring adjustments to the speed of entry to the highway and extra attention to avoid collisions.

**Figure 7:**
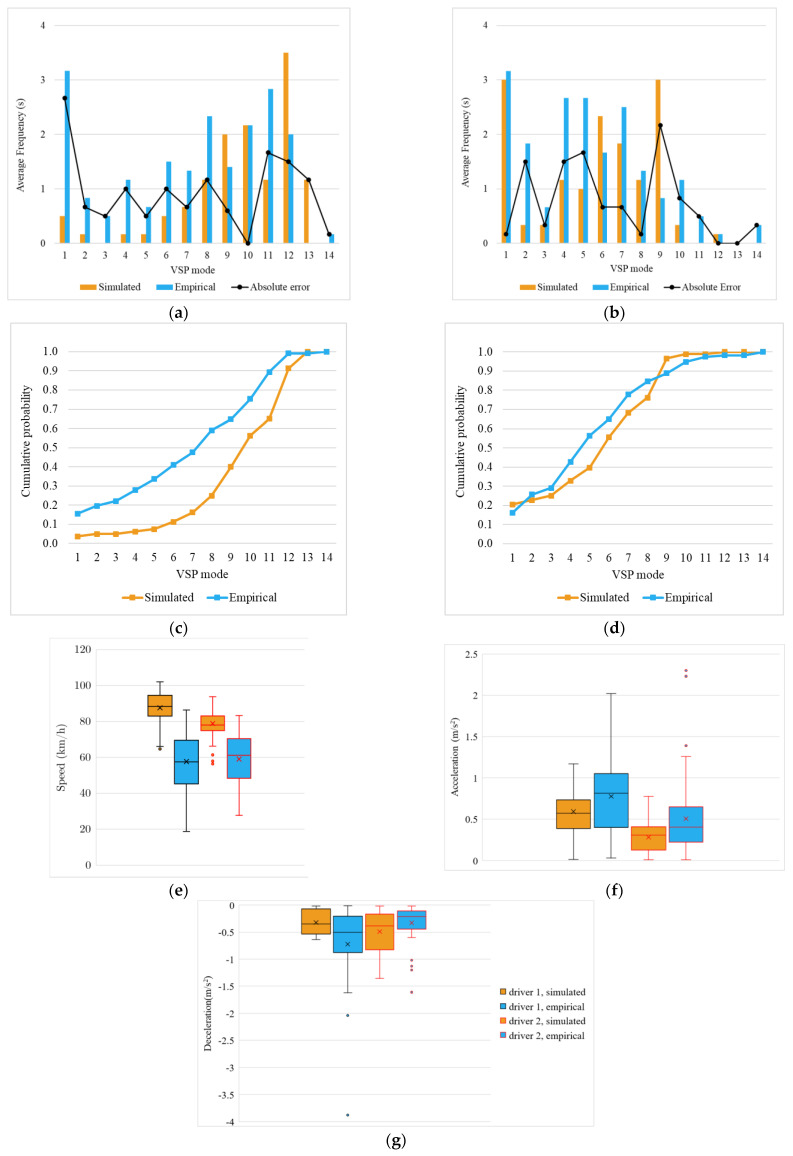
Entering highway event results: average of time spent in each VSP mode and absolute error between simulated and empirical values for Driver 1 (**a**) and Driver 2 (**b**). Cumulative distributions of simulated and empirical VSP modes for Driver 1 (**c**) and Driver 2 (**d**). Box plot for speed (**e**), acceleration (**f**), and deceleration (**g**).

When analysing the graph in Figure 7a, the absolute difference between the empirical and simulated average frequencies of modal bin 1 is evident. The increased traffic volumes seen in the empirical tests can readily account for this disparity. Driver 1 faced difficulties entering the highway due to high traffic flow, prompting the need to slow down in the acceleration lane. The inability to pick up the necessary speed to keep up with the main traffic flow speed on the highway led to abrupt decelerations.

The cumulative probability of the VSP mode being 6 or less (Figure 7c) is 10% for the simulated trips and 40% for the empirical trips, indicating a stark contrast in Driver 1 behaviour on these two types of tests. Driver 2 shows a smaller deviation in the distribution of VSP modes, and the K-S test (Section 4.4) does not reveal a statistically different distribution.

Results suggest that in the simulator tests, the perception of risk and difficulty entering the highway was lower, thus contributing to the higher frequency of high VSP modes on the virtual road. Both participants’ average speeds also confirm this. Figure 7e shows that the driving simulator tests were higher and more stable than those in the real-world environment. The acceleration graphics show less variability in the simulated environment, reinforcing the simulator’s perception of more stable driving.

In this event, the external traffic conditions and the driver’s perception of risk influenced different behaviours for drivers in both scenarios. These behavioural differences in both cases led to an underestimation of previously reported estimated emissions in a simulated environment.

### 4.4. Overall Performance—Probability Distributions

Table 5 summarises the results of the K-S test for all events considering the significance threshold of 5%. Results suggest we reject the null hypothesis, that is, that the frequencies of the VSP modes in the real-world and simulated tests are derived from the same distributions and are indicated by bold cells (*p*-value < 5%). It is observed that in most cases in the urban environment, there are no significant differences in the distribution of the VSP modes. The opposite trend is observed in the highway results.

It may be argued that in an urban environment, there is less freedom for the driver to assume different behaviours. On the other hand, in a highway environment, there is greater freedom to assume different behaviours. Additionally, the perception of risk and potential consequences could lead to greater behavioural differences between the two scenarios.

It is also confirmed that the final output of estimated or measured emissions will not be relevant to validate the simulator for environmental studies. Several cases were identified where different behaviours led to similar emissions results. The relative emissions errors between empirical and simulated data are lower in the highway despite higher differences in driving behaviour. The effect of cancelling errors with the opposite sign is visible at the microscopic level (within each simulated event) and at a complete trip scale through successive under- and overestimations. These results reinforce the need to validate future experiments by following and extending comparison and validation methodologies in a manner similar to this work.

## 5. Conclusions

This paper presented research focused on assessing the reliability of simulators to reproduce vehicle dynamics. In particular, two different experiments (under a driving simulator and in real-world road environments) were conducted based on two scenarios: urban and highway. For the real-world experiment, a probe vehicle was equipped with specific devices to collect engine data as well as traffic-related conditions visual data. The experiments were performed by two volunteers, and more than 18,000 data points from driving experiments were collected. This study’s main goal was to develop an exploratory analysis of potential validation methods for driving simulators for application in environmental studies. For both experiments, emissions were estimated through VSP methodology; thus, the actual parameter under focus is the VSP operating mode of the vehicle (simulated and real).

Several conclusions can be drawn from this validation study:

1. Aligned with the previous literature, drivers agreed that they were less anxious about collisions in the simulator, suggesting that there is a tendency to drive more carelessly in those settings because they were less concerned about the effects of their driving behaviour.
2. The high deceleration values in the simulated environment can also be attributed to the simulator’s brakes operating with much less force than a real car and the lack of motion stimuli.
3. There are several metrics to evaluate the performance of the simulator. However, more than the final output in terms of emissions, it is important to analyse in detail the model’s capability to reproduce driving cycles in terms of the following:
  - Specific traffic events;
  - Kinematic data (speed, acceleration, vehicle specific power distribution);
  - The relative model’s capability to reproduce trends and individual behaviours.

Not being the primary objective of this article due to the limited number of drivers analysed, this exploratory analysis shows that in most cases, the simulator correctly reproduces the driving dynamics associated with microevents in traffic in urban areas. In particular, for the highway scenario, the analysis shows that there may be more variability in the driving behaviour among participants, since contrary to the urban scenario, drivers present small variability in their driving behaviour due to the constraints of the urban environment, which, mostly for safety reasons, do not allow for extreme- or high-speed and acceleration patterns. The difference in observed dynamics on highways suggests that drivers’ adaptability to the traffic context must be considered in further studies. Regarding the estimated emissions, for the urban scenario, the events “turn left without priority” and “give way” presented relative errors of up to almost 35%, while for the highway it was found that data collected from the simulator yielded an underestimation of emissions (up to 20%) for the “entering highway” event. Concerning the total average estimated emissions for all studied events (by route), results suggest that between simulated and real emissions, these were not significantly different, presenting a deviation of approximately 4%, showing that in general, the simulator can capture the vehicle dynamics so that the estimation of environmental impact is reliable.

Future research should also evaluate specific driver driving styles by exploring the effects on the vehicle operation and associated emissions of more driver-related variables, such as pedal position and different gear-shift strategies. Driver factors such as age and driving experience should be examined to understand how driver familiarity with real road conditions affects their performance in a simulator. Additionally, different road traffic and weather conditions should be explored in future experiments. It is also envisaged to assess the driving behaviour in both experiments with feedback warning messages to evaluate changes in the profiles. Although we were only able to provide results for two volunteers, our future research expects to replicate the experiments with more volunteers so that we can enlarge the sample and potentially generalise our results and account for individual variability, reducing the uncertainty in the data. This will certainly enrich the understanding of driving behaviour.

## Figures and Tables

**Table 1:** Critical distances and number of repetitions for each urban and highway event.

Event #			Repetitions—Driver 1		Repetitions—Driver 2	
		Studied Distance (m)	Empirical Simulated		Empirical Simulated	
Urban 1	Right turn with priority	60	19	5	14	5
Urban 2	Left turn without priority	60	13	15	5	11
Urban 3	Right turn after a traffic light	150	8	5	5	5
Urban 4	Left turn after a traffic light	150	6	5	4	5
Urban 5	Right turn after a stop sign	60	7	10	5	7
Urban 6	Left turn after a stop sign	60	17	5	11	5
Urban 7	Give way	60	11	20	5	12
Highway 8	Entering highway	300	6	6	6	6
Highway 9	Exiting highway	300	6	6	6	6
Highway 10	Moving forward	300	9	7	14	14

**Table 2:** Speed, acceleration, and deceleration data from Driver 1 and Driver 2 (simulated results highlighted in orange; empirical results in blue).

	Simulated												Empirical											
	Speed (km/h)																							
	Driver 1						Driver 2						Driver 1						Driver 2					
	Min	Q1	Q2	Q3	Max	Average	Min	Q1	Q2	Q3	Max	Average	Min	Q1	Q2	Q3	Max	Average	Min	Q1	Q2	Q3	Max	Average
Urban 1 Right turn with priority	15.18	20.18	23.73	29.83	46.04	25.38	0.00	19.55	22.80	28.01	41.81	22.00	0.00	13.48	19.00	25.00	38.00	19.52	4.00	12.00	15.00	22.00	33.00	16.82
Urban 2 Left turn without priority	0.00	0.35	10.78	25.57	43.03	13.61	0.00	0.55	11.00	22.43	48.19	13.47	0.00	11.00	17.06	26.10	38.34	17.35	0.00	6.00	19.00	24.00	33.00	16.93
Urban 3 Right turn after a traffic light	0.00	0.00	9.58	24.36	46.07	13.65	0.00	0.00	8.24	28.43	51.43	14.73	0.00	0.00	14.54	21.00	34.00	13.01	0.00	0.00	11.00	19.00	29.00	10.57
Urban 4 Left turn after a traffic light	0.00	0.00	5.64	26.37	43.54	12.29	0.00	0.00	5.85	25.73	37.08	12.04	0.00	0.00	0.00	17.98	39.10	9.25	0.00	0.00	7.00	20.00	31.00	10.38
Urban 5 Right turn after a stop sign	0.00	1.80	14.08	24.49	42.92	14.59	0.00	11.59	19.46	26.71	45.10	18.82	2.92	8.68	18.00	25.00	41.00	17.88	2.00	9.00	15.00	20.00	34.00	15.75
Urban 6 Left turn after a stop sign	0.00	6.96	16.79	25.52	39.65	16.45	0.00	2.46	14.54	24.39	35.55	14.35	0.00	3.00	12.67	21.00	38.41	12.70	0.00	4.00	10.00	18.00	29.00	11.19
Urban 7 Give way	0.00	2.67	14.89	26.81	51.69	15.66	0.00	1.17	14.36	24.89	40.69	14.43	0.00	6.00	16.00	23.00	35.00	15.02	0.00	5.00	19.00	25.00	28.00	15.53
Highway 8 Entering highway	64.72	83.44	88.35	94.22	102.11	87.60	56.37	75.10	78.00	82.92	93.75	78.84	18.72	45.23	57.49	69.59	86.36	57.71	27.76	48.38	61.20	70.34	83.23	59.04
Highway 9 Exiting highway	69.82	84.18	89.17	96.43	112.81	90.80	61.29	69.71	73.44	81.04	86.99	74.76	44.35	66.09	73.67	80.22	86.62	71.56	48.92	70.74	76.73	81.68	88.38	75.67
Highway 10 Moving forward	75.06	86.93	95.58	106.30	117.17	96.44	49.56	66.02	79.11	89.68	109.08	77.87	66.31	81.83	88.27	92.01	101.81	85.87	10.22	33.70	64.87	33.70	92.81	60.02
	Acceleration (m/s^2^)																							
	Driver 1						Driver 2						Driver 1						Driver 2					
	Min	Q1	Q2	Q3	Max	Average	Min	Q1	Q2	Q3	Max	Average	Min	Q1	Q2	Q3	Max	Average	Min	Q1	Q2	Q3	Max	Average
Urban 1 Right turn with priority	0.18	0.64	1.09	1.30	1.47	0.96	0.01	0.44	0.74	0.87	1.50	0.69	0.06	0.47	0.83	1.42	2.50	0.94	0.28	0.28	0.83	1.11	1.67	0.77
Urban 2 Left turn without priority	0.01	0.70	1.28	1.48	1.60	1.08	0.01	0.67	0.98	1.13	1.57	0.88	0.01	0.39	0.83	1.67	3.31	1.06	0.28	0.28	0.56	0.83	1.11	0.61
Urban 3 Right turn after a traffic light	0.01	0.26	0.81	1.24	1.58	0.79	0.02	0.28	0.56	1.07	1.52	0.68	0.01	0.24	0.54	0.83	3.37	0.70	0.28	0.28	0.56	0.90	1.67	0.68
Urban 4 Left turn after a traffic light	0.01	0.30	0.76	1.20	1.57	0.70	0.00	0.19	0.44	0.81	1.34	0.52	0.01	0.35	0.74	1.11	3.17	0.87	0.28	0.42	0.56	0.83	2.50	0.78
Urban 5 Right turn after a stop sign	0.01	0.63	1.32	1.47	1.60	1.08	0.05	0.56	0.88	1.14	1.53	0.85	0.22	0.56	0.93	1.12	1.74	0.88	0.28	0.56	0.69	1.11	1.94	0.86
Urban 6 Left turn after a stop sign	0.00	0.73	1.22	1.45	1.59	1.05	0.07	0.70	1.21	1.50	1.54	1.08	0.04	0.49	0.83	1.35	3.48	0.96	0.28	0.28	0.56	1.11	1.67	0.75
Urban 7 Give way	0.00	0.54	1.23	1.50	1.50	1.02	0.01	0.64	1.23	1.50	1.50	1.05	0.03	0.58	1.06	1.39	2.22	1.05	0.28	0.35	0.69	0.83	0.83	0.73
Highway 8 Entering highway	0.01	0.39	0.57	0.73	1.17	0.59	0.01	0.13	0.30	0.41	0.78	0.28	0.03	0.40	0.81	1.05	2.02	0.78	0.01	0.22	0.40	0.65	2.30	0.51
Highway 9 Exiting highway	0.00	0.12	0.30	0.44	0.94	0.32	0.01	0.18	0.31	0.40	0.58	0.29	0.01	0.06	0.15	0.26	0.47	0.17	0.01	0.11	0.23	0.44	0.90	0.29
Highway 10 Moving forward	0.00	0.09	0.23	0.29	0.33	0.20	0.00	0.18	0.28	0.49	1.04	0.34	0.01	0.14	0.35	0.59	0.90	0.38	0.01	0.11	0.23	0.44	0.90	0.29
	Deceleration (m/s^2^)																							
	Driver 1						Driver 2						Driver 1						Driver 2					
	Min	Q1	Q2	Q3	Max	Average	Min	Q1	Q2	Q3	Max	Average	Min	Q1	Q2	Q3	Max	Average	Min	Q1	Q2	Q3	Max	Average
Urban 1 Right turn with priority	−3.34	−1.79	−0.96	−0.64	−0.20	−1.27	−4.31	−1.75	−1.15	−0.72	−0.35	−1.37	−4.11	−1.60	−1.11	−0.56	−0.02	−1.19	−3.89	−1.67	−1.11	−0.52	−0.28	−1.33
Urban 2 Left turn without priority	−6.06	−2.88	−1.32	−0.57	0.00	−1.74	−4.21	−2.37	−1.26	−0.45	0.00	−1.47	−3.52	−1.67	−0.78	−0.28	−0.01	−1.02	−1.94	−1.32	−0.83	−0.35	−0.28	−0.90
Urban 3 Right turn after a traffic light	−5.35	−1.47	−0.84	−0.38	−0.05	−1.11	−4.11	−2.16	−0.65	−0.20	0.00	−1.20	−3.06	−1.27	−0.56	−0.17	−0.01	−0.78	−2.22	−1.39	−0.83	−0.56	−0.28	−0.97
Urban 4 Left turn after a traffic light	−3.66	−1.24	−0.71	−0.39	0.00	−0.99	−3.08	−0.96	−0.52	−0.24	−0.01	−0.79	−3.41	−1.39	−0.83	−0.41	−0.01	−0.96	−2.78	−1.94	−1.39	−0.56	−0.28	−1.27
Urban 5 Right turn after a stop sign	−4.70	−2.95	−1.57	−0.75	−0.07	−1.83	−4.04	−2.50	−1.11	−0.64	−0.01	−1.53	−2.75	−1.78	−1.25	−0.83	−0.28	−1.33	−2.22	−1.67	−0.83	−0.56	−0.28	−1.07
Urban 6 Left turn after a stop sign	−3.94	−1.93	−1.11	−0.66	−0.10	−1.39	−4.13	−1.90	−0.65	−0.39	−0.01	−1.15	−3.90	−1.52	−1.01	−0.54	−0.03	−1.07	−3.06	−1.81	−1.11	−0.56	−0.28	−1.29
Urban 7 Give way	−5.34	−3.06	−1.26	−0.66	0.00	−1.86	−4.50	−1.84	−0.87	−0.53	0.00	−1.34	−3.06	−1.88	−1.11	−0.71	−0.19	−1.18	−1.94	−1.25	−0.59	−0.28	−0.28	−0.80
Highway 8 Entering highway	−0.64	−0.53	−0.35	−0.07	−0.02	−0.32	−1.36	−0.82	−0.39	−0.17	−0.02	−0.49	−3.88	−0.88	−0.51	−0.20	−0.01	−0.72	−1.61	−0.45	−0.22	−0.11	−0.02	−0.33
Highway 9 Exiting highway	−3.15	−0.91	−0.36	−0.14	−0.02	−0.63	−0.65	−0.2	−0.08	−0.08	−0.01	−0.15	−2.90	−0.88	−0.53	−0.26	−0.02	−0.64	−2.39	−0.88	−0.40	−0.23	−0.02	−0.60
Highway 10 Moving forward	−1.22	−0.80	−0.61	−0.32	−0.01	−0.60	−1.19	−0.50	−0.29	−0.17	−0.01	−0.35	−0.69	−0.26	−0.16	−0.09	−0.01	−0.19	−1.32	−0.55	−0.34	−0.15	−0.01	−0.38

**Table 3:** Average event and total emissions for each type of test and driver, together with the relative error for the empirical value and coefficient between associated emissions (urban scenario).

Event: Urban 1—right turn with priority						
	CO_2_ (g)			NO_x_ (g)		
	Driver 1	Driver 2	Driver 1/Driver 2	Driver 1	Driver 2	Driver 1/Driver 2
Simulated	23.48	23.95	0.98	0.26	0.27	0.96
Empirical	26.5	27.52	0.96	0.3	0.31	0.97
Relative error	−11%	−13%		−13%	−13%	
Event: Urban 2—left turn without priority						
	CO_2_ (g)			NO_x_ (g)		
	Driver 1	Driver 2	Driver 1/Driver 2	Driver 1	Driver 2	Driver 1/Driver 2
Simulated	39.95	35.94	1.11	0.45	0.4	1.13
Empirical	30.85	26.95	1.14	0.35	0.3	1.17
Relative error	29%	33%		29%	33%	
Event: Urban 3—right turn after traffic light						
	CO_2_ (g)			NO_x_ (g)		
	Driver 1	Driver 2	Driver 1/Driver 2	Driver 1	Driver 2	Driver 1/Driver 2
Simulated	83.65	75.87	1.10	0.94	0.85	1.11
Empirical	80.09	96.59	0.83	0.9	1.09	0.83
Relative error	4%	−21%		4%	−22%	
Event: Urban 4—left turn after traffic light						
	CO_2_ (g)			NO_x_ (g)		
	Driver 1	Driver 2	Driver 1/Driver 2	Driver 1	Driver 2	Driver 1/Driver 2
Simulated	90.17	88.76	1.02	1.01	0.99	1.02
Empirical	112.75	97.78	1.15	1.26	1.1	1.15
Relative error	−20%	−9%		−20%	−10%	
Event: Urban 5—right turn after a stop sign						
	CO_2_ (g)			NO_x_ (g)		
	Driver 1	Driver 2	Driver1/Driver2	Driver 1	Driver 2	Driver1/Driver2
Simulated	34.17	28.34	1.21	0.38	0.32	1.19
Empirical	29.17	30.31	0.96	0.33	0.34	0.97
Relative error	17%	−6%		15%	−6%	
Event: Urban 6—left turn after a stop sign						
	CO_2_ (g)			NO_x_ (g)		
	Driver 1	Driver 2	Driver1/Driver2	Driver 1	Driver 2	Driver1/Driver2
Simulated	32.66	35.85	0.91	0.37	0.4	0.93
Empirical	38.19	39.5	0.97	0.43	0.45	0.96
Relative error	−14%	−9%		−14%	−11%	
Event: Urban 7—give way						
	CO_2_ (g)			NO_x_ (g)		
	Driver 1	Driver 2	Driver 1/Driver 2	Driver 1	Driver 2	Driver 1/Driver 2
Simulated	35.12	36.29	0.97	0.4	0.41	0.98
Empirical	33.49	27.72	1.21	0.38	0.32	1.19
Relative error	5%	31%		5%	28%	
Sum of urban events’ average emissions						
	CO_2_ (g)			NO_x_ (g)		
	Driver 1	Driver 2	Driver 1/Driver 2	Driver 1	Driver 2	Driver 1/Driver 2
Simulated	315.72	301.04	1.05	3.55	3.38	1.05
Empirical	348.03	342.8	1.02	3.91	3.87	1.01
Relative error	−9%	−12%		−9%	−13%	

**Table 4:** Average event and total emissions for each type of test and driver, together with the relative error for the empirical value and coefficient between associated emissions (highway scenario).

Event: Highway 8—entering highway						
	CO_2_ (g)			NO_x_ (g)		
	Driver 1	Driver 2	Driver 1/Driver 2	Driver 1	Driver 2	Driver 1/Driver 2
Simulated	64.07	43.24	1.48	0.80	0.50	1.60
Empirical	73.66	54.05	1.36	0.86	0.62	1.39
Relative error	−13%	−20%		−7%	−19%	
Event: Highway 9—exiting highway						
	CO_2_ (g)			NO_x_ (g)		
	Driver 1	Driver 2	Driver 1/Driver 2	Driver 1	Driver 2	Driver 1/Driver 2
Simulated	39.55	43.49	0.91	0.46	0.49	0.94
Empirical	35.31	41.19	0.86	0.40	0.47	0.85
Relative error	12%	6%		15%	4%	
Event: Highway 10—moving forward						
	CO_2_ (g)			NO_x_ (g)		
	Driver 1	Driver 2	Driver 1/Driver 2	Driver 1	Driver 2	Driver 1/Driver 2
Simulated	49.65	43.22	1.15	0.59	0.50	1.18
Empirical	42.73	42.32	1.01	0.50	0.49	1.02
Relative error	16%	2%		18%	2%	
Sum of highway events’ average emissions						
	CO_2_ (g)			NO_x_ (g)		
	Driver 1	Driver 2	Driver 1/Driver 2	Driver 1	Driver 2	Driver 1/Driver 2
Simulated	153.27	129.94	1.18	1.85	1.49	1.24
Empirical	151.70	137.56	1.10	1.76	1.58	1.11
Relative error	1%	−6%		5%	−6%	

**Table 5:** K-S test values.

Event	Driver 1		Driver 2	
	*D-Value*	*D-Critical*	*D-Value*	*D-Critical*
Right turn with priority	0.14	0.21	**0.24**	**0.20**
Left turn without priority	0.07	0.13	0.08	0.19
Right turn after a traffic light	0.11	0.12	0.08	0.12
Left turn after a traffic light	**0.12**	**0.11**	0.12	0.12
Right turn after a stop sign	0.09	0.18	0.16	0.20
Left turn after a stop sign	0.17	0.18	0.17	0.17
Give way	0.09	0.12	**0.20**	**0.17**
Entering highway	**0.34**	**0.20**	0.17	0.19
Exiting highway	**0.35**	**0.21**	**0.37**	**0.20**
Moving forward	**0.28**	**0.19**	**0.15**	**0.14**

## Data Availability

Data will be available upon request.

## Abbreviations

The following abbreviations are used in this manuscript:

GHG	Greenhouse gases
CO_2_	Carbon dioxide
NO_x_	Nitrogen oxides of
HC	Hydrocarbon
CO	Carbon monoxide
PM	Particulate matter
VSP	Vehicle specific power
GNSS	Global Navigation Satellite System
OBD	On-board diagnostics
K-S	Kolmogorov–Smirnov
